# TRIal to slow the Progression Of Diabetes (TRIPOD): study protocol for a randomized controlled trial using wireless technology and incentives

**DOI:** 10.1186/s13063-019-3749-x

**Published:** 2019-11-28

**Authors:** Robyn Su May Lim, Daphne Su Lyn Gardner, Yong Mong Bee, Yin Bun Cheung, Joann Bairavi, Mihir Gandhi, Su-Yen Goh, Emily Tse Lin Ho, Xinyi Lin, Ngiap Chuan Tan, Tunn Lin Tay, Eric Andrew Finkelstein

**Affiliations:** 1 Health Services & Systems Research, Duke-NUS Medical School, 8 College Road, Singapore, 169857 Singapore; 2 Department of Endocrinology, Singapore General Hospital, Outram Road, Singapore, 169608 Singapore; 3 Centre for Quantitative Medicine, Duke-NUS Medical School, 8 College Road, Singapore, 169857 Singapore; 4 Department of Biostatistics, Singapore Clinical Research Institute, 31 Biopolis Way, Singapore, 138669 Singapore; 5 Singapore Institute for Clinical Sciences, A*STAR, 30 Medical Drive, Singapore, 117609 Singapore; 6 Department of Research, SingHealth Polyclinics, 167 Jalan Bukit Merah, Singapore, 150167 Singapore; 7 Department of Endocrinology, Changi General Hospital, 2 Simei Street 3, Singapore, 529889 Singapore

**Keywords:** Diabetes, Smartphone apps, mHealth, Behavior change, Physical activity, Weight monitoring, Blood glucose monitoring, Medication adherence, Financial incentive

## Abstract

**Background:**

The outcomes for those with type 2 diabetes mellitus (T2DM) in Singapore are poor. In this TRIal to slow the Progression Of Diabetes (TRIPOD), we will evaluate the effectiveness and cost-effectiveness of a comprehensive diabetes management package (DMP), with or without a financial incentives program, M-POWER Rewards, in efforts to improve HbA_1c_ levels for individuals with T2DM.

**Methods/design:**

TRIPOD is a randomized, open-label, controlled, multi-center, superiority trial with three parallel arms: (1) usual care only, (2) usual care with DMP, and (3) usual care with DMP plus M-POWER Rewards. A total of 339 adults with sub-optimally controlled T2DM (self-reported HbA_1c_ 7.5–11.0%) will be block randomized according to a 1:1:1 allocation ratio to the three arms. The primary outcome is mean change in HbA_1c_ level at Month 12 from baseline. Secondary outcomes include mean change in HbA_1c_ level at Months 6, 18, and 24; mean changes at Months 6, 12, 18, and 24 in weight, blood pressure, and self-reported physical activity, weight monitoring, blood glucose monitoring, medication adherence, diabetes self-management, sleep quality, work productivity and daily activity impairment, and health utility index; and proportion of participants initiating insulin treatment by Months 6, 12, 18, and 24. Incremental cost-effectiveness ratios will be computed based on costs per improvement in HbA_1c_ at Month 12 and converted to cost per quality-adjusted life year gained.

**Discussion:**

The TRIPOD study will present insights about the long-term cost-effectiveness and financial viability of the interventions and the potential for integrating within usual care.

**Trial registration:**

ClinicalTrials.gov, NCT03800680. Registered on 11 January 2019.

## Background

### Rationale

Diabetes prevalence has been steadily increasing worldwide and is projected to cost the global economy up to US$ 2.5 trillion, or 2.2% of the global gross domestic product, in 2030 alone [1, 2]. In Singapore, forecasts suggest that, without interventions, the lifetime risk of developing type 2 diabetes mellitus (T2DM) will be one in two by 2050 with a concomitant increase in total economic costs to US$ 1.9 billion in 2050 alone due to increased morbidity resulting from the condition [3, 4].

Systematic reviews and meta-analyses strongly support the effectiveness of various lifestyle interventions in reducing blood sugar levels—as measured by glycated hemoglobin (HbA_1c_)—and body weight, two key outcomes that have been associated with lowering diabetes-associated health risks [5, 6], especially among patients with sub-optimal glycemic control [7]. Successful interventions include diabetes self-management education [8], physical activity targets [9], weight management [10], blood glucose self-monitoring [11], medication adherence [12], and personal health coaching [13].

Although behavioral interventions are often impractical to administer in primary care and community settings due to limited resources and infrequent patient interaction, technological advances now enable us to deliver lifestyle interventions through mobile health (mHealth) apps and devices, providing low-cost, highly adaptable, and scalable solutions. We hypothesize that a comprehensive mHealth program incorporating key lifestyle intervention strategies, with or without financial incentives for healthy behaviors, could offer a scalable, cost-effective, and potentially cost-saving approach to address Singapore’s diabetes epidemic.

In a previous feasibility study, we evaluated a proprietary lifestyle management program, GlycoLeap (designed and produced by KKT Technology Pte. Ltd., Holmusk, Singapore), which was originally developed for adults with T2DM in Singapore [14]. The 24-week GlycoLeap program consisted of a comprehensive T2DM educational curriculum delivered online and the Glyco smartphone app, through which users logged and monitored their blood glucose levels, weight, meals, and physical activity and received personal health coaching by accredited dietitians. Although we observed statistically significant improvements in HbA_1c_ (− 1.3 percentage points, *P* < 0.001) and weight reduction (2.3% reduction from baseline weight, *P* < 0.001), the proportion of participants meeting recommended weekly self-care process targets declined throughout the 24-week period for all evaluated components, potentially attenuating the longer term benefits of sustained lifestyle management [14]. To address this concern, we have developed a rewards program based on behavioral economic theory to complement GlycoLeap. The rewards program leverages present bias, loss aversion, and habit formation and thus offers the potential for lasting benefits.

In this randomized controlled trial, we test whether adding a comprehensive diabetes management package (DMP), with or without a financial incentives program (M-POWER Rewards), can improve HbA_1c_ levels and other health outcomes of individuals with T2DM. One of the health outcomes we will assess is change in body weight, as weight reduction in overweight (body mass index, BMI ≥ 23 kg/m^2^) individuals with T2DM is associated with decreases in HbA_1c_ levels [15]. The DMP comprises the Glyco app and the M-POWER smartphone app and also includes the following features: diabetes self-management education, physical activity tracking, weight management, blood glucose self-monitoring, medication adherence tracking, and personal health coaching. The M-POWER app serves as a one-stop portal for participants to monitor their own diabetes self-management processes and also incorporates social norms as a behavioral tool by comparing individual self-care processes with those of others (descriptive norms) and displaying congratulatory or motivating messages for satisfactory or inadequate performance, respectively (injunctive norms) [16]. Social norms have been tested in behavioral health interventions and shown to encourage healthier food consumption [17–19]. Additionally, the app includes a gamified element to harness innate motivation by displaying an individual’s current and best streaks (number of consecutive weeks where the target has been met) for all components. The M-POWER Rewards program offers rewards in the form of M-Points for both short-term processes (weekly activity targets) and longer term health outcomes (biannual HbA_1c_ and weight reduction goals). Our incentive strategy leverages loss aversion by disbursing M-Points as rebates for approved medical expenditures, including expenses typically incurred at usual care visits [20]. This rebate strategy could potentially encourage greater clinic appointment attendance and lower barriers to purchasing prescribed diabetes medication, glucometer consumables for sustained self-monitoring, and other diabetes-care consumables.

Because of the high costs involved in treating people with chronic conditions, employers, insurers, and governments all have a financial incentive to contain the chronic disease epidemic. Therefore, these third-party payers have shown a willingness to invest in some level of prevention and treatment efforts. If this study demonstrates cost-effectiveness, or even cost savings, we believe that third-party payers will be inclined to fund or subsidize the adoption of our intervention and incentive program in the primary care or community setting.

### Objectives and hypotheses

#### Primary outcome and hypothesis

The objective of this study is to determine whether complementing usual care with the DMP, with or without financial rewards (M-POWER Rewards) can improve mean HbA_1c_ levels at Month 12 (primary endpoint) of individuals with T2DM.

We hypothesize that between baseline (date of HbA_1c_ test blood sample collection and randomization) and Month 12, mean improvements in HbA_1c_ level will be greatest in the DMP plus M-POWER Rewards arm, followed by DMP alone, followed by usual care.

#### Secondary outcomes and hypotheses

We will test for differences between groups in mean change in HbA_1c_ levels from baseline at Months 6, 18, and 24. We will also test mean differences between groups for changes in weight and blood pressure, proportion of participants who progress to insulin, self-reported physical activity, weight monitoring, blood glucose monitoring, medication adherence, diabetes self-management, sleep quality, work productivity, daily activity impairment, and health utility index at all four time points (Months 6, 12, 18, and 24). All outcomes will be tested with the hypothesis that improvements will be greatest in the DMP plus M-POWER arm, followed by DMP, followed by usual care.

We will also test potential effect modifiers, including duration of T2DM, baseline HbA_1c_ level, previous or existing experience with mobile apps to manage health conditions, and number of diabetes medications. We hypothesize that those with a longer duration of T2DM, no experience with health management apps, and more diabetes medications will be less successful in improving the primary outcome.

#### Secondary objectives

The secondary objectives are to determine net costs and incremental cost-effectiveness ratios (ICERs) of each intervention arm. ICERs will be calculated based on costs per improvement in HbA_1c_ at Month 12 and converted to cost per quality-adjusted life year (QALY) gained. Using established benchmarks for cost-effectiveness [21, 22], we hypothesize that DMP alone will be cost-effective compared to usual care and, despite higher implementation cost, DMP plus M-POWER will be incrementally cost-effective relative to DMP alone.

## Methods/design

### Study design

**Fig. 1:**
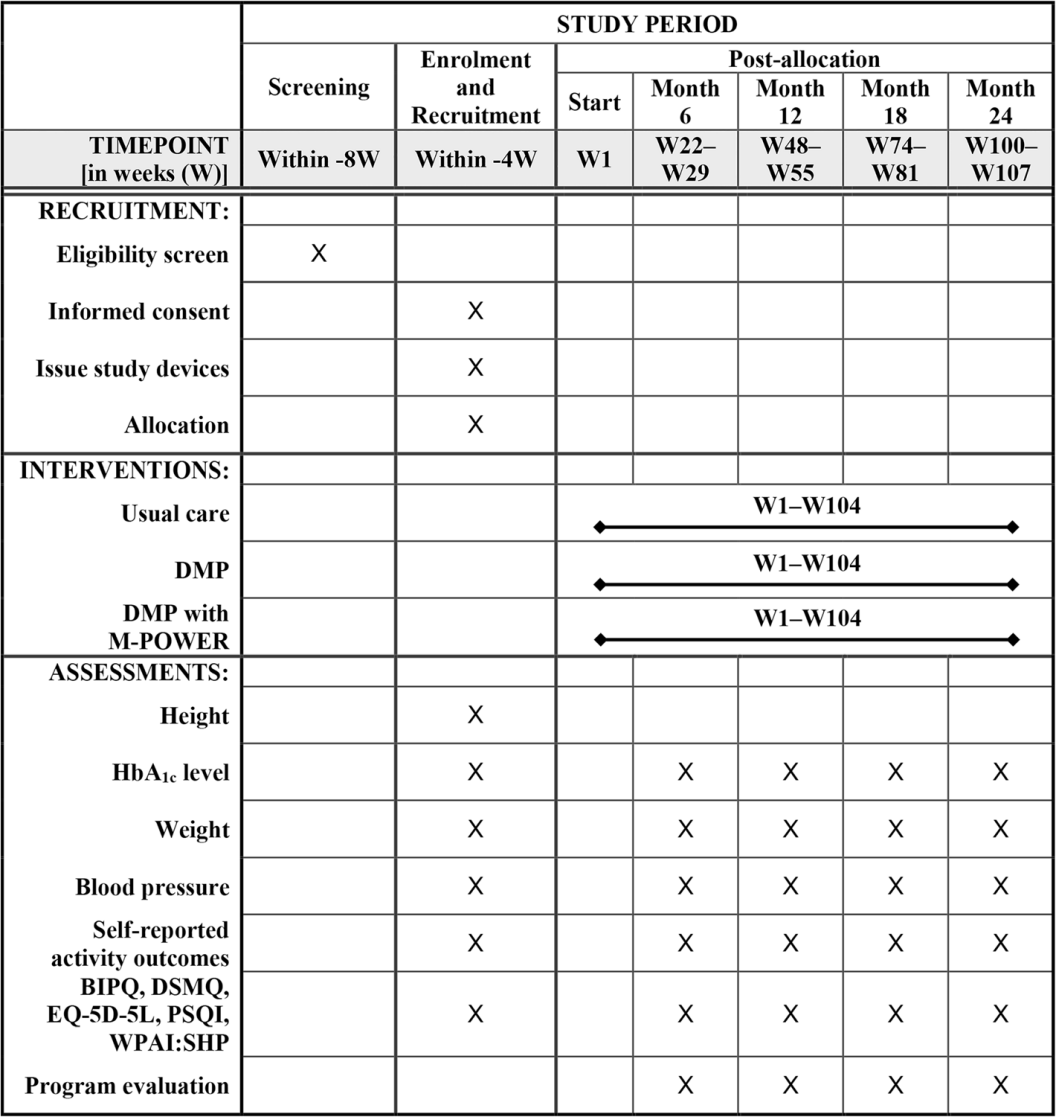
SPIRIT figure of enrollment, interventions, and assessments. *DMP* diabetes management package, *HbA*_*1c*_ glycated hemoglobin, *BIPQ* Brief Illness Perception Questionnaire, *DSMQ* Diabetes Self-Management Questionnaire, *EQ-5D-5 L* EuroQoL 5-dimension 5-level health utility index, *PSQI* Pittsburgh Sleep Quality Index, *WPAI:SHP* Work Productivity and Activity Impairment: Specific Health Problem instrument

The TRIal to slow the Progression Of Diabetes (TRIPOD) is designed as a randomized, open-label, controlled, multi-center, superiority trial with three parallel arms. A total of 339 adults with sub-optimally controlled T2DM will be block randomized according to a 1:1:1 allocation ratio to the three arms. The study intervention will last for 24 months (104 weeks), and the primary outcome is the mean change in HbA_1c_ level at Month 12 from baseline as measured by blood tests. This protocol conforms to the Standard Protocol Items: Recommendations for Interventional Trials (SPIRIT) guidelines see (Additional file 1 and Fig. 1).

### Study setting and eligibility criteria

Adults (aged 21 to 70) with sub-optimally controlled T2DM from 11 Singapore Health Services (SingHealth) referral sites (two specialist diabetes centers and nine polyclinics) will refer themselves directly to the study team from Duke-NUS Medical School, who will manage and execute all study-related procedures, including the study visits (i.e., training sessions and follow-up assessments), at Duke-NUS Medical School or other study venues, if available. SingHealth is Singapore’s largest public healthcare group and is in a collaborative academic medicine partnership with Duke-NUS Medical School. Collectively, the SingHealth referral sites serve both low- and high-income patients, with all Singaporean citizens and permanent residents entitled to government subsidies. All eligibility criteria will be self-declared.

#### Inclusion criteria

Individuals will be included who:

1. Have been diagnosed with T2DM with sub-optimal diabetes control as defined by an HbA_1c_ level between 7.5 and 11.0% (inclusive) at the most recent test taken within the past 3 calendar months
2. Are not on insulin
3. Are on at least one oral glucose-lowering drug
4. Are aged 21 to 70 at last birthday
5. Are Singapore citizens or permanent residents
6. Are able to read, write, and communicate in English
7. Own a personal smartphone and are comfortable with using apps.

#### Exclusion criteria

Patients will be excluded if they:

1. Are pregnant or lactating
2. Have ahistory of chronic kidney disease
3. Have undergone dialysis for treatment of kidney failure
4. Have a history of cardiovascular disease
5. Have a history of stroke
6. Have a history of blood diseases
7. Have a history of chronic liver disease
8. Have undergone chemotherapy, radiation therapy, or immunotherapy for cancer treatment in the past 5 years
9. Have undergone blood transfusion in the past 3 months
10. Are taking systemic corticosteroids
11. Have a history of bariatric surgery or extensive bowel resection
12. Have had any major surgery in the past year
13. Are unable to walk up 10 stair steps (individual steps, not floors) without stopping/difficulty.

#### Conditional eligibility criterion

As the DMP has a physical activity component, patients will complete the Physical Activity Readiness Questionnaire (PAR-Q) [23] to detect individuals who may be at risk if they increase their physical activity. If patients answer “Yes” to any PAR-Q question, they will be required to consult their physician and provide an approval note from the physician to be able to participate in the study.

### Participant timeline and study arms

Interested patients will be directed to take an online screener questionnaire to assess their eligibility to join the study. All eligible patients will be invited to complete an online baseline questionnaire and attend a training session conducted by the study team where they will be briefed on the study and will sign an informed consent form (i.e., enrollment). During the training session, participants will have their baseline anthropometric measurements taken by the study team, their blood drawn by trained phlebotomists for HbA_1c_ tests, and their arm allocation revealed to them (i.e., recruitment). All patients will receive arm-specific participant booklets containing information about the study design, timeline, visits, recommended activities, interventions, and payouts. Participants in both intervention arms will receive the DMP along with training on how to use the study devices and apps. In addition, participants in the DMP plus M-POWER Rewards arm will be given details about the M-POWER Rewards program and will sign a participant oath declaring that all activity data that they will provide solely represents their own efforts without attempts to cheat. Research has revealed that such oaths might reduce the probability that individuals will engage in dishonest behavior [24].

Participants will remain in their respective study arms throughout the 24-month (104-week) study. Follow-up questionnaires and assessments will be administered within 8-week window periods at Month 6 (Weeks 22–29), Month 12 (Weeks 48–55), Month 18 (Weeks 74–81), and Month 24 (Weeks 100–107). All follow-up assessments will be conducted at Duke-NUS Medical School (or other study venues, if available) where anthropometric measurements will be taken and blood samples collected for HbA_1c_ tests.

**Fig. 2:**
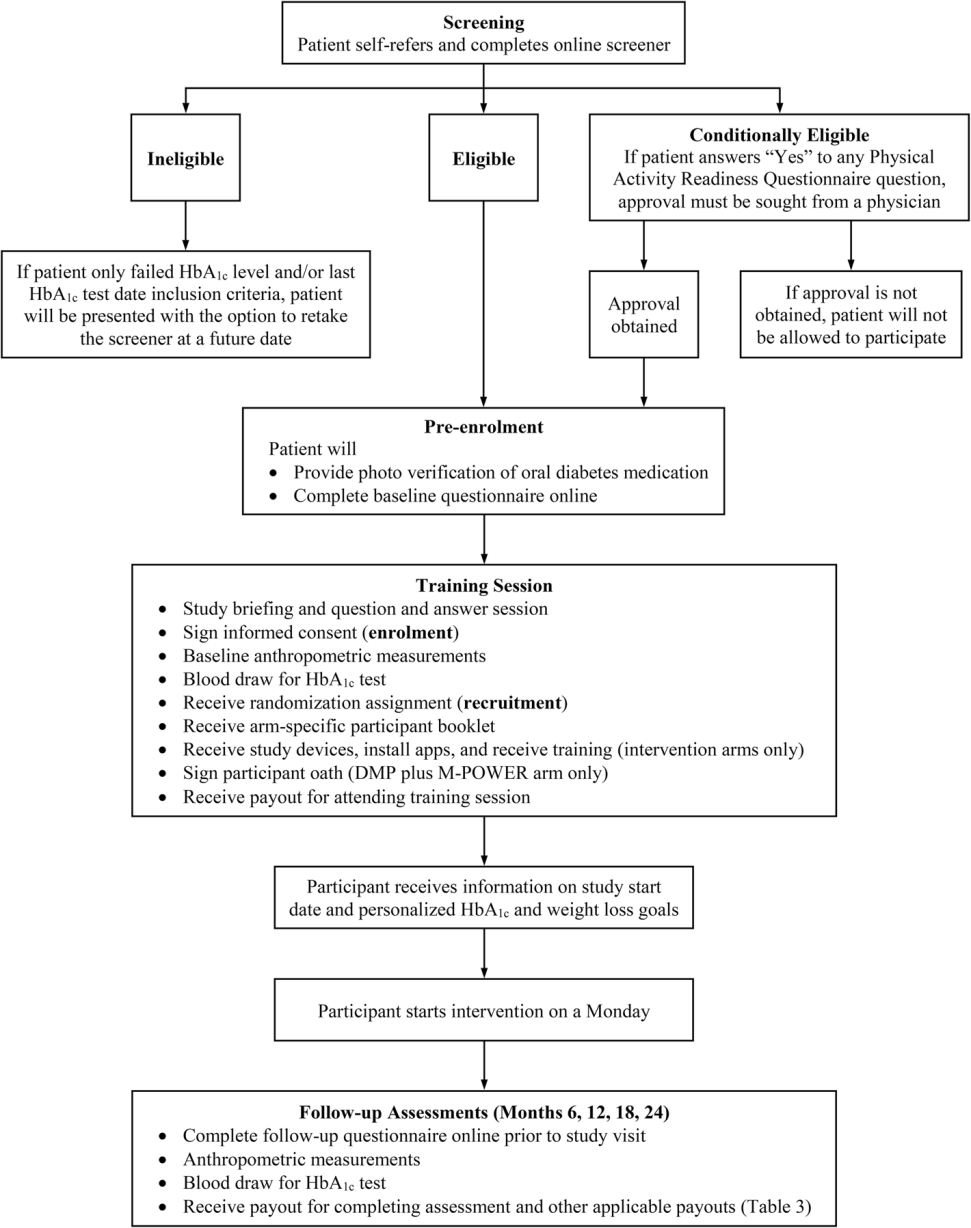
TRIPOD participant flow diagram

Figure 2 illustrates the flow diagram for study participants.

#### Arm 1: usual care

Participants in the usual care arm will continue to receive usual care at their diabetes clinics throughout the study. As this study seeks to assess the effectiveness and cost-effectiveness of complementing usual care with the DMP, with or without M-POWER Rewards, the choice of usual care as a comparator is appropriate. In order to better identify the effect of the DMP and M-POWER Rewards interventions, we will present the same diabetes self-management recommendations to participants in all arms. Usual care arm participants will be encouraged to perform the following recommended activities meant to improve glycemic control during the course of the study:

1. Learn more about diabetes self-management
2. Engage in at least 150 min of moderate-to-vigorous exercise per week
3. Monitor weight at least once a week and aim to achieve a healthy BMI (< 23 kg/m^2^)
4. Monitor blood glucose with a glucometer at least three times a week on separate days and aim to achieve post-meal (2 hours after meals) readings within 4.0–10.0 mmol/L
5. Take diabetes medication as prescribed during usual care.

Participants in the usual care arm will also be encouraged to achieve two health goals by the end of the 24-month (104-week) study: (1) achieve reduction of ≥ 1 percentage point in HbA_1c_ level from baseline and (2) lose ≥ 5% of initial body weight at baseline for those with BMI ≥ 23 kg/m^2^ at baseline or, for those with BMI < 23 kg/m^2^ at baseline, maintain a healthy weight.

#### Arm 2: diabetes management package (DMP)

Similarly to those in the usual care arm, participants in the DMP intervention arm will continue to receive usual care at their diabetes clinics and will be encouraged to achieve the same HbA_1c_ level reduction and weight loss goals as those provided to the usual care arm. In addition, as part of the DMP, participants will receive the following apps, devices, and recommendations:

1. *M-POWER smartphone app*. Designed and created for this study, the M-POWER app will serve as a one-stop portal for participants to monitor their diabetes self-management activities and progress throughout the study. The app syncs and displays relevant data from all apps and study device accounts provided to participants. Participants will also be able to view study-relevant medical information, specifically HbA_1c_ test results and weight measured at assessments, and personalized HbA_1c_ level reduction and weight loss goals on the M-POWER app. Personal progress for all activities will be illustrated in graphical form for easy comprehension. Besides displaying individual progress, the app will compare personal results with the average results of all participants within their respective arm (descriptive norms), along with congratulatory or motivating messages for satisfactory or inadequate performance, respectively (injunctive norms). The participant’s current and best streaks (number of consecutive weeks where the target has been met) for all components will also be shown on the app.
2. *GlycoLeap digital lifestyle and education program*. This 24-week education and behavior change program is designed and produced by KKT Technology Pte. Ltd. (Holmusk, Singapore) for patients with T2DM in Singapore. It is delivered through a smartphone app, Glyco, and comprises interactive, educational lessons and quizzes and human health coaching.
  *(a) Lessons and quizzes*. Participants are given a diabetes self-management education curriculum in the form of 24 lessons accessible from the Glyco app. At the end of each lesson, participants will be presented with a quiz containing multiple choice questions to test their understanding and retention of lesson content. Quiz scores ≥ 80% confer a passing grade, and the scores of the first passed attempts or the latest results will be displayed on the M-POWER app. Participants can revisit the health lessons and retake the quizzes at any time throughout the study.
  *(b) Personal health coaching and support*. Participants will receive personalized advice, guidance, and positive motivation from a team of health coaches through the Glyco app during the 24-week GlycoLeap program. Having a personal coach introduces accountability, which is an important driver of behavior change. All health coaches are qualified and accredited dietitians in Singapore. To improve the value of coaching, health coaches will provide personalized coaching based on data that participants have input in the Glyco app (see the following paragraphs), including food logs. Participants can create food logs by using the Glyco app to take photographs of their meals. These photos will be reviewed and rated based on nutritional quality and portion sizing by the health coaches.
3. *Pedometer for physical activity tracking*. Participants will be provided with a Fitbit™ pedometer (Fitbit™ Inc., San Francisco, CA, USA) and access to an anonymous Fitbit™ account that will be created for the study, and recommended to engage in at least 150 min of moderate-to-vigorous physical activity (MVPA) while targeting for at least 420 Fitbit™ active minutes weekly (see Discussion for explanation). Participants will also be encouraged to sync their Fitbit™ devices with their Fitbit™ study accounts wirelessly at least once a week. Active minutes from participants’ Fitbit™ accounts will be displayed on the M-POWER app.
4. *Weighing scale for weight monitoring*. Participants will be provided with a basic bathroom weighing scale with a digital display and recommended to weigh themselves at least once a week and log their weight measurements manually on the Glyco app. Weight logs that have been entered on the Glyco app, along with personalized weight goals, will be displayed on the M-POWER app.
5. *Glucometer for blood glucose monitoring*. Participants will be provided with a CONTOUR™ PLUS ONE glucometer (Ascensia Diabetes Care Holdings AG, Basel, Switzerland) and recommended to take at least three post-meal (2 h after meals) measurements on separate days a week and aim to achieve readings within 4.0–10.0 mmol/L. Participants will be encouraged to upload their data by wirelessly syncing their glucometers with the Glyco app at least once a week for updated glucometer readings to be displayed on the M-POWER app.
6. *Pill tracker for medication adherence*. Participants will be provided with an RX Cap™ pill tracker (DoseSmart™ Inc., San Francisco, CA, USA) and access to an anonymous DoseSmart™ account that will be created for the study, and recommended to use the pill tracker with the oral glucose-lowering drug that has been assigned for the study and to take their medication as prescribed. For medication assignment by the study team, metformin will be the first choice, as most T2DM patients on an oral glucose-lowering drug will be expected to have been prescribed metformin. If participants are not on metformin, the oral glucose-lowering drug with the most frequent dosing will be assigned instead. Participants will be encouraged to upload their data by wirelessly syncing their pill trackers with their DoseSmart™ study account at least once a week for their medication adherence to be displayed on the M-POWER app.

#### Arm 3: DMP and M-POWER Rewards program (DMP plus M-POWER Rewards)

Participants in the DMP plus M-POWER intervention arm will also continue to receive usual care at their diabetes clinics and the same HbA_1c_ reduction and weight loss goals as those provided to the other two arms. In addition to the DMP intervention, participants will be entitled to the M-POWER Rewards program. Our rewards strategy takes advantage of rebates to address loss aversion and a mix of near-term and longer term goals to address present bias. Participants can earn up to 1000 M-Points (1 M-Point is equivalent to 1 Singapore dollar [SGD1]) over the 2-year study period for performing specific care processes according to study recommendations (Table 1) and for achieving HbA_1c_ and weight loss goals (Table 2). The rewards scheme offers more M-Points for meeting targets that are more challenging but more likely to reap better health benefits. M-Points can be redeemed in the form of financial rebates for approved non-inpatient, healthcare-related expenses incurred during the study period. Approved expenses include clinic and outpatient visits, laboratory tests, medications, medical devices (e.g., glucometers), consumables (e.g., glucose test strips), and other approved health-monitoring devices (e.g., physical activity trackers, weighing scales). To redeem their accrued M-Points, participants will submit photos of their receipts via the M-POWER app to the study team for approval. Participants may view their earned, redeemed, and balance M-Points on the M-POWER app. All redemptions will be issued in cash.

**Table 1:** M-POWER rewards scheme for performing recommended activities

Category and app/ device	Criteria	M-Points		Terms and conditions
		Award	Max over 104 weeks	
Bonus	All arm 3 participants will be credited with a bonus at the start of their study	8 bonus M-Points	8	Can only be claimed if participants earn ≥ 12 M-Points throughout the study
Health literacy, Glyco app	Complete the GlycoLeap lesson quizzes and achieve a score of 80% or higher to pass	1 M-Point per quiz passed	24	Quizzes are taken on the Glyco app and can be retaken until a passing grade is achieved
Weight monitoring, basic weighing scale and Glyco app	Weekly weigh-ins	1 M-Point per week	104	Weigh-ins must be self-reported weekly through the Glyco app
Physical activity, Fitbit pedometer and Fitbit app	Achieving Fitbit’s active minutes	3 M-Points if ≥ 420 Fitbit active minutes per week 2 M-Points if 350–419 Fitbit active minutes per week	312	Data must be uploaded via the Fitbit app
Blood glucose monitoring, Ascensia Diabetes Care CONTOUR PLUS ONE glucometer and Glyco app	Weekly post-meal glucose measurements (taken 2 h after each meal) must be within range 4.0–10.0 mmol/L	2 M-Points per week if ≥ 3 post-meal measurements (with each measurement taken on a separate day) per week between 4.0–10.0 mmol/L	208	Data must be uploaded via the Glyco app
Medication adherence, DoseSmart pill tracker and DoseSmart app	Compliance is considered met when the medication is taken based on both prescribed number of times per day and prescribed time of the day (if applicable)	1 M-Point per week if 100% compliant to medication schedule within the week	104	Data must be uploaded via the DoseSmart app

**Table 2:** M-POWER Rewards scheme for achieving HbA_1c_ and weight loss goals

Bonus M-Points awarded at each follow-up assessment	Criteria for earning M-Points	
	If BMI ≥ 23 kg/m^2^ at baseline	If BMI < 23 kg/m^2^ at baseline
60 bonus M-Points	Attain a ≥ 1.0 percentage point decrease in HbA_1c_ level from baseline or Lose ≥ 5% of weight from baseline weight	Attain a ≥ 1.0 percentage point decrease in HbA_1c_ level from baseline
30 bonus M-Points	Maintain HbA_1c_ level or attain up to 1.0 percentage point decrease in HbA_1c_ level from baseline or Maintain weight or lose up to 5% weight from baseline weight	Maintain HbA_1c_ level or attain up to 1.0 percentage point decrease in HbA_1c_ level from baseline

### Outcome measures

#### Primary outcome

The primary outcome is mean change in HbA_1c_ level at Month 12 from baseline. Decreases in HbA_1c_ level have been associated with risk reductions in diabetes-related clinical complications or mortality, and diabetes randomized trials frequently use mean reduction in HbA_1c_ level as a study outcome [5, 25].

#### Secondary outcomes

The secondary outcomes are:

1. Mean change in HbA_1c_ level at Months 6, 18, and 24 from baseline
2. Mean change in weight at Months 6, 12, 18, and 24 from baseline
3. Mean change in systolic and diastolic blood pressure at Months 6, 12, 18, and 24 from baseline
4. Proportion of participants who had insulin treatment initiated by their diabetes care physician by Months 6, 12, 18, and 24
5. Mean change in self-reported physical activity at Months 6, 12, 18, and 24 from baseline as assessed using the Global Physical Activity Questionnaire (GPAQ) [26]
6. Mean change in self-reported weight monitoring at Months 6, 12, 18, and 24 from baseline as assessed by frequency of self-weighing
7. Mean change in self-reported blood glucose monitoring at Months 6, 12, 18, and 24 from baseline as assessed by frequency of self-testing
8. Mean change in self-reported medication adherence at Months 6, 12, 18, and 24 from baseline as assessed by frequency of taking diabetes medications as prescribed
9. Mean change in diabetes self-management at Months 6, 12, 18, and 24 from baseline as assessed using the Diabetes Self-Management Questionnaire (DSMQ) [27]
10. Mean change in sleep quality at Months 6, 12, 18, and 24 from baseline as assessed using the Pittsburgh Sleep Quality Index (PSQI) [28]
11. Mean change in work productivity and daily activity impairment at Months 6, 12, 18, and 24 from baseline as assessed using a modified Work Productivity and Activity Impairment: Specific Health Problem instrument (WPAI:SHP) [29]
12. Mean change in health utility index at Months 6, 12, 18, and 24 from baseline as assessed using the 5-level EQ-5D instrument [30]
13. ICERs based on HbA_1c_ level, determined by calculating the incremental cost per percentage point unit reduction in HbA_1c_ level at Month 12 (primary endpoint)
14. ICERs based on QALYs, determined by converting ICER based on HbA_1c_ level into incremental cost per QALY gained.

### Sample size

Data from Bilger et al. [31] showed a standard deviation (SD) of HbA_1c_ levels averaged across usual care and intervention groups being 1.2% at both baseline and after a 6-month intervention and a correlation of 0.4 between the two time points. We assume an SD of 1.2% in HbA_1c_ at Month 12 (primary endpoint) and a correlation of 0.2 between baseline and Month 12 measurements. In order to detect a mean difference of 0.5% between the usual care and DMP arms and 0.5% between the DMP and DMP plus financial incentive arms, with analysis of covariance (ANCOVA) adjustment for baseline HbA_1c_ and multiplicity adjustment by the closed testing procedure [32], a sample size of 90 per group is needed to have 80% power at a two-sided 5% familywise type 1 error rate. To allow for 20% attrition at Month 12, we will recruit 113 patients per arm, or 339 in total.

### Randomization

Participants will be randomized according to a 1:1:1 allocation ratio to the three arms, using stratified randomization with random permuted blocks within strata. The block size will be determined by a statistician generating the randomization list and will not be disclosed to the investigators and other study team members who have contact with study participants. Three stratification factors will be used: diabetes center (specialist clinic or polyclinic), gender, and dichotomized HbA_1c_ level at baseline (7.5–9.2% or 9.3–11.0%). For allocation concealment, sequentially numbered, opaque, and sealed randomization envelopes will be used for the randomization assignment for all participants.

### Participant recruitment, retention, withdrawal, discontinuation, and adverse event reporting

#### Participant recruitment

Eleven SingHealth referral sites (two specialist diabetes centers and nine polyclinics) will serve as referral sites for this study. Patients attending regular diabetes care at these referral sites will be recruited through various advertising avenues at the referral sites, newspaper advertising, and online sites. All recruitment materials will briefly explain the study design, list the key eligibility requirements, and direct interested participants to the study website that contains additional information about the study. The website will also contain contact information of the study team and serve as a means for interested patients to connect with the study team via a contact form. Interested patients may assess their eligibility by completing an online eligibility screener on the website, and prospective participants will be requested to provide their contact details and consent to be contacted. If the patient did not report having an HbA_1c_ level within the eligibility range or having taken an HbA_1c_ test within the past 3 calendar months, the patient will be presented with the option to have the study team arrange for the patient to retake the screener at a future date after the next regular, clinic-scheduled HbA_1c_ test has been taken. If a prospective participant answers “Yes” to any PAR-Q question [23], he/she will be required to provide an approval note from a physician to be able to participate in the study (conditional eligibility). Patients who answered “No” to all questions will be deemed physically fit to engage in physical activity as recommended in the DMP. The study team will contact all prospective participants to provide additional details about the study, answer any questions that patients might have, obtain photographs of the patients’ oral glucose-lowering drug to verify their medication and clinic, set up a unique personal link to complete the baseline questionnaire, and schedule a baseline training session. Prospective participants will attend training sessions at Duke-NUS Medical School (or other study venues, if available) where they will be briefed on the study, enrolled, measured for baseline values, recruited, and taught how to use the study devices, apps, and the M-POWER Rewards program. As an incentive to join the study and for compensation for their time, successfully recruited participants will receive SGD10 in cash at the end of the training session. Figure 2 illustrates the flow diagram for participants.

#### Participant retention

Participants will receive SGD30 at each assessment for successfully completing assessments within their respective window periods. Participants in the usual care arm and the DMP incentive arm will receive SGD150 and SGD70, respectively, as forms of “fairness” payouts for not receiving the DMP and/or incentives entitled to other arms. Table 3 lists the participant payouts by arm.

**Table 3:** Participant payouts

Type of payout	Condition	Payouts		
		Arm 1 (Usual care)	Arm 2 (DMP)	Arm 3 (DMP plus M-POWER)
Attending training session	Attend the training session	SGD10	SGD10	SGD10
Completing assessments	Complete assessments at Months 6, 12, 18, and 24 within their respective window periods	SGD30 per assessment	SGD30 per assessment	SGD30 per assessment
Study device data upload	Each device (pedometer, glucometer, and pill tracker) needs to contain at least one entry within the first 7 calendar days from the first Monday (inclusive) of the month to receive payouts for that device for that month The data must be uploaded successfully	NA	SGD2 per device per month^a^	SGD2 per device per month^a^
M-POWER Rewards	As per M-POWER Rewards scheme (Tables 1 and 2)	NA	NA	Max 1000 M-Points (SGD1000) over 104 weeks
Fairness payout	Complete both Month 12 and Month 24 assessments within their respective window periods Awarded upon completion of the Month 24 assessment	SGD150 for entire study^b^	SGD70 for entire study^c^	NA

^a^SGD6 per month for three devices (pedometer, glucometer, and pill tracker) in total

^b^For not receiving DMP, incentives for study device data upload, and M-POWER Rewards

^c^For not receiving M-POWER Rewards

Participant burden will be minimized by limiting participant in-person visits to five sessions (one baseline and four follow-up assessments), although additional visits may be necessary to disburse cash payouts or provide replacement devices. Malfunctioned or misplaced devices will be replaced at a subsidized rate (depending on budget) to enable continued participation of participants in the intervention arms.

#### Participant withdrawal and discontinuation

Participants are free to withdraw from the study at any time by informing the study team or the investigators of their decision to withdraw. Data that has been collected until the time of their withdrawal will be stored and analyzed. Participants may be discontinued from the study due to one or more of the following reasons:

1. They become pregnant.
2. Upon voluntarily informing their doctor that they are participating in this study, their doctor decides that continuing participation could be harmful and informs us in the process.
3. They fail to follow the instructions of the study team or investigators.

Participants who develop any of the exclusion criteria 2–13 during the course of the study will not be withdrawn from the study unless they choose to withdraw voluntarily. There are no concomitant care or other interventions that will be prohibited during the study.

#### Adverse event reporting

Before commencing the study, participants will be informed that they should report the occurrence of any potential adverse events during the course of the study to the study team. The study team will ask participants about potential adverse events during the follow-up assessments. In the event that the study team is informed of any serious adverse event (SAE), the principal investigator will notify the SingHealth Centralized Institutional Review Board (CIRB) by submitting the SAE Reporting Form within the stipulated time frame. The notifying and reporting requirements depend on the severity, nature and causality of the event, and specific procedures as delineated by SingHealth will be followed [33]. SAEs will also be reported to the National University of Singapore Institutional Review Board (NUS-IRB).

### Data collection

#### Survey data

The screener questionnaire will be administered online via the study website. The baseline and Months 6, 12, 18, and 24 questionnaires will be provided to participants through unique, personal Qualtrics™ (Qualtrics International Inc., Provo, UT, USA) links sent via email. All questionnaires will contain questions to assess secondary outcomes, including the DSMQ, GPAQ, PSQI, a modified WPAI:SHP, and 5-level EQ-5D survey instruments, as well as the Brief Illness Perception Questionnaire (BIPQ), which has been validated in English for diabetes [34, 35]. Additionally, the baseline questionnaire includes questions on socioeconomic characteristics, while the follow-up questionnaires include questions on program evaluation.

#### Health outcomes

Measurements for health outcomes will be recorded at the training session (baseline) and at the Months 6, 12, 18, and 24 follow-up assessments conducted at Duke-NUS Medical School (or other study venues, if available). HbA_1c_ tests will be conducted using the high-performance liquid chromatographic method via the VARIANT™ II TURBO Hemoglobin Testing System (Bio-Rad Laboratories Inc., Hercules, CA, USA) on the blood samples collected at all study visits. No blood samples will be stored after the HbA_1c_ tests have been completed. Height (Seca 217 Mobile Stadiometer; Seca GmbH, Hamburg, Germany) will only be measured at baseline. Weight (Seca 869 Mobile Floor Scale; Seca GmbH, Hamburg, Germany) and blood pressure (Welch-Allyn 420 Spot Vital Signs BP Monitor; Welch Allyn, Skaneateles Falls, NY, USA) will be measured at all study visits. For height and weight, duplicate measurements will be recorded, along with a third measurement if the first two readings are unequal. For systolic and diastolic blood pressure, measurements will be taken after a period of rest of at least 5 min. Three readings will be taken with 3-min intervals of rest between each measurement, and the average of the last two readings will be used.

#### Study devices and apps

Participants in the two intervention arms will wirelessly sync data from their pedometer, glucometer, and pill tracker with their anonymous Fitbit™, Glyco, and DoseSmart™ study accounts, respectively. Weight logs and quiz scores captured on the Glyco app will be stored in participants’ Glyco accounts. In order to display updated participant activities and M-Points on the M-POWER app, the study platform will pull data from all the anonymous study accounts on a daily basis via API and automatically calculate and award M-Points daily.

#### Data for sensitivity analysis and cost-effectiveness analysis

Medical data such as HbA_1c_ test results taken during usual care at the clinics and diabetes medications prescribed and purchased, including insulin initiation dates, will be obtained from the medical records and pharmacy bills of all participants. HbA_1c_ test results from these medical records will be used in sensitivity analyses. Billing data from inpatient, outpatient, pharmacy, and emergency departments will be collected to estimate net costs and cost offsets for the cost-effectiveness analysis. The costs of program delivery will be determined by capturing data on all relevant (non-sunk) labor costs, materials and supplies, contracted services (including costs for GlycoLeap), and M-POWER Rewards payouts.

### Data management and monitoring

During the study, all data will be stored on secure servers at Duke-NUS Medical School and will be backed up daily. All data containing personal identifiers will be encrypted with password protection. All physical research data, including consent forms, data entry forms, and password-protected portable hard disk drives containing backup data, will be stored in locked cabinets at Duke-NUS Medical School. Only de-identified data will be shared with statisticians for data analysis, and only the investigators and study team directly involved with the study will have access to the data. The research data will be kept for at least 10 years after research completion and securely destroyed upon the publication of all pertinent research studies or reports.

A Data and Safety Monitoring Board (DSMB) with members who are external to the study team and independent of the study and sponsor has been established to oversee matters on participant data security, participant safety, and implementation fidelity. Members consist of two consultant endocrinologists, a biostatistician, and the head of Duke-NUS Medical School’s information technology department. The DSMB charter will be made available upon request. No termination guidelines or rules have been defined for this study.

This study is subject to reviews and/or audits by the SingHealth Research Quality Assurance Unit. These reviews or audits may be conducted routinely, triggered by the SingHealth CIRB, or upon an investigator’s request.

#### Survey data

Each completed screener questionnaire attempt will generate a unique ID that will be traceable to individual prospective participants. Similarly, each personal Qualtrics™ link for baseline and follow-up questionnaires will contain the participant’s unique study ID, ensuring that we can differentiate survey entries completed by different participants. Data validation will be implemented to ensure that responses are provided for all questions and that questionnaire entries are only flagged as complete after the participant reaches the end of the questionnaire. For the baseline questionnaire, if a participant completes the same questionnaire more than once by mistake, each entry will be recorded separately and timestamped, allowing us to assess the multiple entries. Only one entry from each participant will be used for data analysis. For the follow-up questionnaires, participants will only be permitted to complete each questionnaire once. Survey data from all questionnaires will be downloaded by the study team regularly and checked for completion and any errors or inconsistencies.

#### Health outcomes

Measurements taken at the study visits will be recorded on hardcopy printouts. All data will be converted into electronic data using double data entry by two different individuals and verified for consistency. Any discrepancies will be resolved by referring to source documents. At the study visits, phlebotomists will verify participants’ identities before drawing blood and will label blood samples with the participants’ study IDs only. HbA_1c_ results will not contain any personal identifiers and will be shared with the study team after each batch of blood samples is processed.

#### Study devices and apps

To encourage participants in the incentive arms to engage with the study components, weekly inactivity notifications that list the components that they have not been engaging with will be emailed to participants. Participants who are not engaging with the study components will be contacted by the study team, who will offer their assistance and ensure that participants are not experiencing problems with the devices or apps. Study team members will also note those who indicate that they do not wish to perform any activity(s) and will not contact these participants for the purpose of troubleshooting. At each follow-up assessment, study team members will also verify that participants are using the devices as instructed and respond to any queries that participants might have on usage of the devices or apps. Besides the aforementioned measures to reduce data loss, participants in both intervention arms will be incentivized to upload their data: participants will be given SGD2 per device per month for syncing their pedometers, glucometers, and pill trackers with their anonymous study accounts see (Table 3).

As participants in the DMP plus M-POWER arm may be tempted to cheat in order to earn more M-Points, they will sign a participant oath at the end of the training session and each time they collect their M-Points redemption payouts. This might help to improve data validity, as such oaths could reduce the likelihood of cheating [24]. The study team will also manage and back up data from the devices and apps and regularly check the data for inconsistencies that may suggest cheating.

### Statistical methods

#### Preliminary descriptive analyses

Preliminary descriptive analyses will be performed, and the patterns of missing data/drop-out rate in each arm will be examined. The statistician conducting the analysis will be blinded to the arm assignments when conducting preliminary descriptive analyses. The arm assignments will be disclosed only after the final database lock. A detailed statistical analysis plan will be prepared before the final database lock.

#### Primary analysis

Mean change in HbA_1c_ level at Month 12 is the primary outcome. The primary analysis will be performed on a modified intention-to-treat population, including participants who have both baseline and Month 12 HbA_1c_ level data. A linear regression model with HbA_1c_ level at Month 12 as the dependent variable and an intercept, HbA_1c_ level at baseline (continuous variable), indicator variables for participants who received DMP alone and participants who received DMP plus M-POWER, and indicator variables for stratification factors (gender and diabetes center) as independent variables will be performed. Using this model, a test for the global null hypothesis of all three arms having equal mean HbA_1c_ level at Month 12 will be performed (Null Hypothesis 1: Coefficient of DMP plus M-POWER = Coefficient of DMP = 0), followed by tests for three pairwise hypotheses, comparing mean HbA1c level at Month 12 in DMP alone vs. usual care (Null Hypothesis 2: Coefficient of DMP = 0), DMP plus M-POWER vs. usual care (Null Hypothesis 3: Coefficient of DMP plus M-POWER = 0), and DMP plus M-POWER vs. DMP alone (Null Hypothesis 4: Coefficient of DMP plus M-POWER = Coefficient of DMP). We will also conduct a sensitivity analysis with further adjustment for dichotomized HbA1c levels at baseline (7.5–9.2% vs. 9.3–11.0%). The differences in the primary outcome between study arms will be presented along with corresponding 95% confidence intervals. Following the closed testing procedure [32] for controlling for multiple comparisons involving three groups, only if tests of both the global null hypothesis and a pairwise null hypothesis reach statistical significance at the 0.05 level will the pairwise null hypothesis be rejected.

If there are substantially different drop-out rates or different drop-out patterns among the three arms, a general linear model for repeated measures will be performed for the primary analysis. This model simultaneously models HbA_1c_ level at baseline, Month 6, and Month 12 as the dependent variables and includes interactions between indicator variables for DMP alone and Month 6 visit, DMP plus M-POWER and Month 6 visit, DMP alone and Month 12 visit, and DMP plus M-POWER and Month 12 visit as independent variables. The model will also adjust for visit (indicator variable for the Month 6 visit and indicator variable for the Month 12 visit) and randomization stratification variables (gender and diabetes center). An unstructured matrix will be used to model the residual variance-covariance structure within participant. The model does not include main effect terms for the intervention variables, and thus it constrains the estimated group means of baseline HbA_1c_ levels to be identical across the three randomized groups. This model specification helps control for the variation in baseline HbA_1c_ level arising by chance among the randomized groups. Using this model, a test for the global null hypothesis of all three arms having equal mean HbA_1c_ level at Month 12 will be performed (Null Hypothesis 1: Coefficient of interaction between indicator of DMP alone and indicator of Month 12 visit = Coefficient of interaction between indicator of DMP plus M-POWER and indicator of Month 12 visit = 0), followed by tests for three pairwise hypotheses, comparing mean HbA_1c_ level at Month 12 in DMP alone vs. usual care (Null Hypothesis 2: Coefficient of interaction between indicator of DMP alone and indicator of Month 12 visit = 0), DMP plus M-POWER vs. usual care (Null Hypothesis 3: Coefficient of interaction between indicator of DMP plus M-POWER and indicator of Month 12 visit = 0), and DMP plus M-POWER vs. DMP alone (Null Hypothesis 4: Coefficient of interaction between indicator of DMP alone and indicator of Month 12 visit = Coefficient of interaction between indicator of DMP plus M-POWER and indicator of Month 12 visit). If the missing data patterns do not necessitate using a general linear model for repeated measures as a primary analysis, this analysis will be conducted as a sensitivity analysis.

#### Secondary effectiveness analyses

##### Quantitative outcomes

Secondary quantitative outcomes (weight, blood pressure, GPAQ total physical activity score, weight monitoring frequency, blood glucose monitoring frequency, diabetes medication adherence frequency, DSMQ sum score, global PSQI score, percent overall work impairment and percent activity impairment due to diabetes and related health problems using a modified WPAI:SHP, health utility index using 5-level EQ-5D) will be analyzed using a similar strategy as applied to the primary outcome. A linear regression model will be used to model the secondary quantitative outcome as the dependent variable and an intercept, the outcome at baseline (quantitative variable), indicator variables for participants who received DMP alone and participants who received DMP plus M-POWER, and indicator variables for stratification factors (gender, diabetes center, and dichotomized HbA_1c_ level at baseline) as independent variables. Additional analyses will also be performed to evaluate the intervention effects that account for insulin progression/medication changes and other potential effect modifiers, mediators, covariates, and program engagement metrics.

##### Binary outcomes

Secondary binary outcomes (e.g., proportion of participants who had insulin treatment initiated by their diabetes care physician) will be analyzed using a generalized linear model with a log link function and binomial distribution (log-binomial regression model). The model will include the following as independent variables: an intercept, indicator variables for participants who received DMP alone and participants who received DMP plus M-POWER, and indicator variables for stratification factors (gender, diabetes center, and dichotomized HbA_1c_ level at baseline).

#### Secondary cost-effectiveness analyses

The net cost and cost-effectiveness analysis will be performed from a third-party payer’s perspective using an activity-based costing approach which the principal investigator has performed in many prior studies [36–38]. Using this approach, the costs of program delivery will be determined by capturing data on all non-sunk labor costs, materials and supplies, contracted services (including costs for GlycoLeap), and M-POWER Rewards payouts. Billing data from inpatient, outpatient, pharmacy, and emergency departments will be collected. We will compute the net costs of each arm and the incremental cost per unit reduction in HbA_1c_ at Month 12 (primary endpoint) as compared to the next most costly intervention. We will also compute the incremental cost per QALY gained based on published studies/models that quantify the relationship between reductions in HbA_1c_ at Month 12 and QALYs gained. Results will be compared to established benchmarks for cost-effectiveness and those of other interventions aimed at reducing HbA_1c_ levels. As a secondary analysis, we will also quantify incremental costs per QALY gained based on the 5-level EQ-5D health utility index captured at Month 12 through questionnaires and assumptions about the duration of any benefits attained during the study. One-way and *n*-way sensitivity analyses and cost-effectiveness acceptability curves that graphically present the probability that each intervention arm is incrementally cost-effective (and potentially cost-saving) for a range of willingness-to-pay metrics that a decision maker may consider will also be presented.

## Discussion

This study evaluates the effectiveness, costs, and cost-effectiveness of a novel, comprehensive lifestyle management package and incentive program targeting two key factors for diabetes-associated health risks [5, 6]. To ensure that our objectives are met, it is imperative that participants understand the study’s aims, design, diabetes care process recommendations, health outcome goals, and the M-POWER Rewards scheme. During the training session, the study team will present briefing slides to thoroughly explain the study and give interested patients an opportunity to clear their doubts through a question-and-answer session. Key information will also be present in the consent form and participant booklet that will be issued to all participants. For participants in the intervention arms, crucial information about the process targets, health goals, and the M-POWER Rewards scheme can be found in the M-POWER app and participant booklet. On the M-POWER app, the targets for each process and personalized health goals will be displayed on the component’s respective progress tab. Participants in the DMP plus M-POWER arm should not confuse the incentives for completing assessments and syncing their data with rewards for care processes and outcomes. We do not foresee this to be a major concern, as incentives for care processes and outcomes are awarded in the form of redeemable M-Points, and the M-Points earned from satisfying each care process target or outcome goal will be clearly reflected on the M-POWER app.

Besides clearly presenting study targets, we have to ascertain their viability. Through testing the Fitbit™ devices, we have discovered that Fitbit™ overestimates their active minutes, which are determined through their proprietary algorithms, as these active minutes appear to include bouts of activity that are less than 3 metabolic equivalents of task (METs). Through our test data, we established that 420 Fitbit™ active minutes is a reasonable and attainable weekly target for participants in the intervention arms (DMP and DMP plus M-POWER arms) if they engage in at least 150 min of MVPA each week.

We have considered the possibility that participants in the DMP plus M-POWER arm may be tempted to cheat and have included oath signing to reduce the likelihood of cheating [24]. This practice has been applied to other financial incentive studies that we have conducted [31, 39, 40]. The study team can perform quality checks to assess for potential cheating cases. For physical activity, we may review the distribution of pedometer step data to identify any improbable or aberrant activity. For blood glucose monitoring, the study team may compare glucometer readings with HbA_1c_ levels to identify any possible inconsistencies. However, cheating will not impact our primary analysis, which is based on HbA_1c_ tests conducted at the study visits. For medication adherence, since participants will be able to change their medication dosing frequency and time windows through their DoseSmart™ accounts, medication prescriptions and bills can be assessed for verification. We do not anticipate much cheating for weight monitoring, as the process target (log weight once a week) is not tied to the weight input and is fairly easy to achieve.

Randomized controlled trials assessing quality improvement strategies for diabetes care, including patient self-management and financial incentives, typically do not test interventions and conduct follow-up assessments up to 24 months, making our study one of the longest in terms of intervention and assessment duration [7, 25]. If one or more arms prove successful, we will evaluate even longer term health outcomes up to 3 years post-intervention (i.e., up to 5 years from baseline). If the DMP plus M-POWER arm demonstrates effectiveness, our study will be among the first T2DM lifestyle management randomized controlled trials utilizing a financial incentive strategy to report statistically significant HbA_1c_ improvement, which was not observed in previous financial incentive trials that assessed HbA_1c_ [31, 41, 42]. Furthermore, if shown to be cost-effective, this study will equip us with insights about the long-term financial viability of the interventions to present to policy makers, since we will be evaluating cost-effectiveness from the third-party payer’s perspective and the potential for integrating with usual care. By evaluating potential effect modifiers or mediating factors, we can identify patient sub-populations who will benefit most from the intervention, allowing for a more targeted approach in the primary care or community setting. Finally, this study will provide valuable information to assist in shaping future interventions and incentive programs for chronic disease management.

### Trial status

As of 18 September 2019, the ethics-approved study protocol is version 6, dated 31 July 2019. Recruitment is anticipated to commence in October 2019 and be completed around July 2020.

## Supplementary information

**Additional file 1.** SPIRIT 2013 checklist.

## Data Availability

Study documents (e.g., informed consent form) and de-identified datasets arising from this study will be made available upon reasonable requests addressed to the corresponding author.

## Abbreviations

ANCOVA: Analysis of covariance
BIPQ: Brief Illness Perception Questionnaire
CIRB: SingHealth Centralized Institutional Review Board
DMP: Diabetes management package
DSMQ: Diabetes Self-Management Questionnaire
GPAQ: Global Physical Activity Questionnaire
HbA_1c_: Glycated hemoglobin
ICER: Incremental cost-effectiveness ratio
MET: Metabolic equivalent of task
mHealth: Mobile health
MVPA: Moderate-to-vigorous physical activity
PAR-Q: Physical Activity Readiness Questionnaire
PSQI: Pittsburgh Sleep Quality Index
QALY: Quality-adjusted life year
SAE: Serious adverse event
SD: Standard deviation
SingHealth: Singapore Health Services
SPIRIT: Standard Protocol Items: Recommendations for Interventional Trials
T2DM: Type 2 diabetes mellitus
TRIPOD: TRIal to slow the Progression Of Diabetes
WPAI:SHP: Work Productivity and Activity Impairment: Specific Health Problem
